# Reversal of Conditioned Food Aversion Using a Cognitive Intervention: A Sham-Controlled, Randomized, Parallel Study

**DOI:** 10.3390/nu15234962

**Published:** 2023-11-29

**Authors:** Adoracion Nieto, Dan M. Livovsky, Fernando Azpiroz

**Affiliations:** 1 Digestive System Research Unit, University Hospital Vall d’Hebron, 08035 Barcelona, Spaindanlivo@szmc.org.il (D.M.L.); 2 Departament de Medicina, Universitat Autònoma de Barcelona, Bellaterra, 08193 Cerdanyola del Vallès, Spain; 3 Centro de Investigación Biomédica en Red de Enfermedades Hepáticas y Digestivas (Ciberehd), Instituto de Salud Carlos III, 28029 Madrid, Spain; 4 Digestive Diseases Institute, Shaare Zedek Medical Center, Faculty of Medicine, Hebrew University of Jerusalem, Jerusalem 9103102, Israel

**Keywords:** Pavlovian conditioning, aversive conditioning, cognitive intervention, eating behaviour, digestive sensations, postprandial symptoms, digestive wellbeing, food valence

## Abstract

Background: Aversive conditioning weakens the gratifying value of a comfort meal. The aim was to determine the effect of a cognitive intervention to reverse aversive conditioning and restore hedonic postprandial response. Methods: This was a randomized, sham-controlled, single-blind, parallel study that was conducted on 12 healthy women (*n* = 6 in each group). The reward value of a comfort meal was measured on different days: at initial exposure, after aversive conditioning (administration of the same meal with a masked fat overload on the previous day) and after a cognitive intervention (disclosing the aversive conditioning paradigm in the test group vs. no explanation in the control group). The primary outcome, digestive wellbeing, was determined using graded scales at regular intervals before and after ingestion. Results: At initial exposure, the comfort meal produced a rewarding experience that was impaired using aversive conditioning; upon re-exposure to the original meal, the cognitive intervention increased meal wanting and liking; improved digestive wellbeing and mood; tended to reduce postprandial satiety, bloating/fullness; and abolished discomfort/pain, thereby restoring the hedonic value of the comfort meal. By contrast, sham intervention had no effects, and the postprandial sensations remained like the responses to the offending meal. Conclusion: In this proof-of-concept study, we demonstrate that in healthy women, a mild, short-term acquired aversion to a comfort meal can be reversed using a cognitive intervention. ClinicalTrials.gov ID: NCT05897411.

## 1. Introduction

Ingestion of a meal induces sensations that persist during the postprandial period in parallel to digestion, and these sensations are key to ingestive behaviour [1]. The postprandial experience is determined by conditions related to the meal and to the eater. Under normal conditions, a comfort meal induces a pleasant experience but may induce aversive sensations in predisposed individuals. For instance, when one becomes ill after consuming a meal, there is a propensity to blame that meal as the cause of the illness, with a hedonic shift from positive to negative. Hence, conditioned food aversion is a learned association between a particular food and negative experience, such that the food becomes aversive [2]. We recently showed that a negative digestive sensation coupled to a comfort meal deteriorates the postprandial hedonic response to subsequent ingestion [3].

We hypothesized that acquired food aversion may be reversed using deconditioning. In this context, the term deconditioning refers to reversal of previously conditioned behaviours, i.e., unlearning aversive conditioning [4,5]. To test our hypothesis, we designed a pilot proof-of-concept study in which we first induced aversion to an originally comfort meal by associative learning, and then used this model to test the effect of a cognitive intervention.

Functional gastrointestinal disorders, the most prevalent diagnoses in gastroenterology, represent a spectrum of conditions characterized by altered interaction between the gut and the brain. This group of disorders is distinguished by gastrointestinal symptoms related to various potential mechanisms, including disturbances in gastrointestinal motility, heightened visceral sensitivity (i.e., visceral hypersensitivity), altered mucosal and immune functions, alterations in gut microbiota, and modifications in central nervous system (CNS) processing of gut-derived stimuli [6]. Specifically, patients with functional gastrointestinal disorders frequently relate their symptoms to meals, and it has been proposed that their origin may be related to aversive conditioning from previous experiences [5,7,8,9]; unlearning aversive conditioning, if proven effective, could become a relevant treatment strategy for these clinical conditions.

## 2. Material and Methods

### 2.1. Experimental Design

A single-blind, sham-controlled, randomized parallel study investigating the impact of a cognitive intervention on the aversive response to a meal was conducted from January to May 2023.

Participants were informed that the study aimed to assess how meal composition influenced their eating experience, and that various hummus recipes with different ingredients would be tested. Participants were randomly assigned to the cognitive intervention group or the control group by means of a computerized random sequence generator. First, both groups underwent aversive conditioning, and, subsequently, the effects of the cognitive intervention were compared to a sham intervention. The research adhered to the Declaration of Helsinki and was conducted in accordance with a preapproved protocol by the Vall d’Hebron Institutional Review Board (Comitè d’Ètica d’Investigació Clinica, Vall d’Hebron Institut de Recerca; protocol PR(AG)338/2016N approved on 28 October 2016, and revised on 19 February 2021), and all were informed and provided consent before the commencement of any study procedures. ClinicalTrials.gov registration number NCT05897411.

### 2.2. Participants

Twelve healthy women (six per group) without obesity and weight stable were enrolled in this study. Participants were recruited through public advertising, and only those who met the entry criteria (see below) were chosen for enrolment. For this proof-of-concept study, we exclusively enrolled female participants to maintain uniformity and align with recent data indicating that women, in comparison to men, exhibit greater sensitivity to conditions influencing the experience after the meal [10]. Candidates were not enrolled in case of chronic health conditions, antecedents of digestive complaints, prior abdominal surgeries (other than appendectomies or hernia repairs), regular medication use (other than occasional NSAIDs and antihistamines), alcohol abuse, recreational drug use, history of anosmia, ageusia, body mass index above 30 kg/m^2^, or any form current dieting of selective eating (e.g., vegetarianism). Only candidates that liked hummus were enrolled in this study. Candidates filled out a standard abdominal symptom questionnaire (no symptoms rated above 2 on a scale of 0 to 10 were allowed), the Hospital Anxiety and Depression Scale (HAD; scores below 7 on the anxiety or depression subscales were required [11]); Dutch Eating Behaviour Questionnaire (DEBQ; thresholds for emotional eating < 2.83, external eating < 3.5, and restrained eating < 3.0 were required [12]) and the Physical Anhedonia Scale (PAS). The experiments were performed on days 5–15 of the menstrual cycle (follicular phase).

A previous study demonstrated a decrease in postprandial satisfaction through aversive conditioning (the area under the curve for digestive wellbeing changed by −186 ± 68 score × min in the test group vs. 16 ± 19 score × min in the control group) [3]. Based on these data, we determined that four participants in each group would be needed to observe changes in the primary outcome with 90% power and a 5% significance threshold. For this study, six participants per group were included, and power calculations were conducted using GPower 3.1 software [13].

### 2.3. General Procedure

During the study period (4 days), participants were asked to refrain from intense exercising and to consume a typical evening meal every night, consisting of 100 g of chicken, 50 g of rice, 50 g of white bread, and 1 apple (providing 503 kcal, 7 g of fat, 82 g of carbohydrates, and 30 g of protein). After an overnight fast, they were instructed to consume a standard breakfast consisting of 200 mL of coffee with milk (semi-skimmed) and a sandwich made with 50 g of white bread, 30 g of ham, and 40 g of cheese (providing 338 kcal, 11 g fat, 38 g carbohydrates, and 24 g protein) four hours before presenting to the lab for each study session. All participants stated that they adhered to the protocol. Digestive sensations were assessed at regular intervals during fast (referred to as the pre-ingestion period), during meal consumption itself (the ingestion period), and during the 60 min following the end of the meal (the postprandial period).

### 2.4. Interventions

#### 2.4.1. Probe Meal

The meal was served at 20 °C and comprised 200 g hummus (Hummus Classic, Ametller Origen, Barcelona, Spain), 26.7 g toast (108 kcal; 1.2 g fat, 20.3 g carbohydrates, 3.2 g protein, Mini Tostas, Bimbo, Barcelona, Spain), and 120 mL mineral water. Following a stepwise procedure, the meal was administered, divided into four equal servings, with each serving presented every 180 s. Each portion consisted of 50 g of hummus along with 6.6 g of toast, which was placed on a platter. Following each serving, 60 s were designated for participants to evaluate their digestive sensations (as described below). The entire ingestion process lasted for 16 min, and participants could consume the 120 millilitres of water at their own pace throughout the ingestion period. Two recipes of hummus were administered, either with regular fat content (292 kcal; 16 g fat, 109 g carbohydrates, 17 g protein or with masked fat overload by addition of 31.3 g of lard (Mont-Palau, Costa Brava Mediterranean Foods, Girona, Spain). The hummus (regular or high-fat) was either coloured using 1.9 g of a pink colourant that is soluble in fat and is devoid of odour and flavour (Decora, Karma, Salerno, Italy) or non-coloured. To match the lipid content of the coloured meal, the hummus on days 1 and 4 were supplemented with 1.9 g of sunflower oil. The fat content of the hummus and the amount of food to be served were decided upon after a series of initial feasibility studies.

#### 2.4.2. Experimental Paradigm

**Figure 1:**
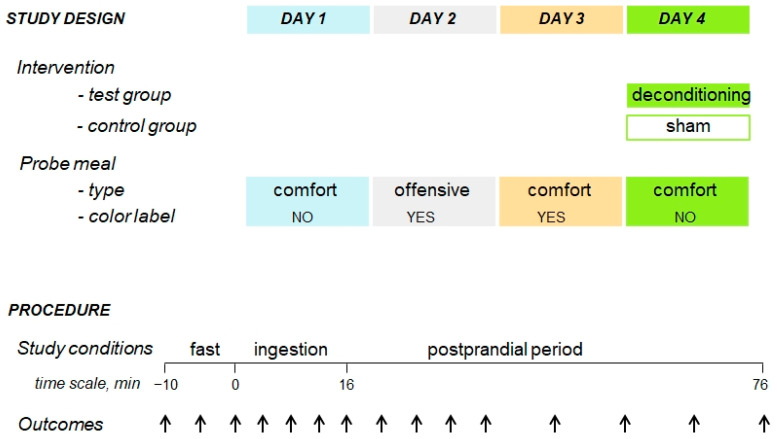
Experimental procedure and design. A sham-controlled, parallel, randomized, blind study was performed, measuring the responses to meal ingestion on four consecutive days. On day 1, a comfort meal was administered. On day 2, an offensive meal (the same meal with a masked fat overload and colour additive) was administered. On day 3, the original comfort meal with the colour additive was administered, to detect the effect of aversive conditioning by previous exposure to the offending meal. On day 4, a cognitive intervention was performed (disclosing the aversive conditioning paradigm in the test group, but not in the control group) and the comfort meal was subsequently retested. Each study day digestive sensations were measured by means of scales during the pre-ingestion period, during consumption, and following ingestion of the probe meal. The arrows at the bottom of the image represent the time points at which the sensations (outcomes) were measured.

Participants completed four experiments conducted on consecutive days, as follows: First day: a comfort meal (tasty hummus with regular fat content and non-coloured) was administered. Second day: an offensive meal (high-fat coloured hummus) was administered to induce aversive conditioning. Third day: proof of aversive conditioning by administration of a coloured hummus with regular fat content. Fourth day: a cognitive intervention was performed in the test group, but not in the control group, and the initial comfort meal (regular-fat, non-coloured hummus) was administered afterwards. The cognitive intervention disclosed the experimental paradigm as follows: participants in the test group were informed that on the 2nd study day, the meal was modified with a hidden fat overload (fat addition not affecting taste but impairing its reward value) plus a colour additive, while on the 3rd study day, only the colorant was added to the original comfort meal (Figure 1). The effect of the intervention on the sensation of digestive wellbeing was the primary outcome, and it was measured as the change in the area under the curve (AUC) from aversive conditioning to the AUC postintervention (i.e., day 4 minus day 3) in the cognitive intervention group against the control group.

### 2.5. Outcome Measures: Hedonic and Homeostatic Sensations

We employed five scales of 10 cm with gradations from −5 to +5 in order to assess the following aspects: (a) meal wanting (ranging from impossible to eager), (b) meal liking (from very disagreeable to very agreeable), (c) hunger/satiety (ranging from extremely hungry to completely satiated), (d) digestive wellbeing (varying from extremely unpleasant sensation to extremely pleasant sensation), and (e) mood (ranging from negative to positive). Scales also of 10 cm but graded from 0 (not at all) to 10 (very much) were used to assess for (f) abdominal bloating–fullness, (g) discomfort–pain, and (h) nausea. Meal wanting was assessed both at the presentation of each serving (inquiring about the desire to consume that portion) and at the conclusion of the entire meal (inquiring about the desire for another portion). Meal liking was evaluated after eating each portion (how much did you like it). The other scales were evaluated (a) before meal ingestion, at 5 min intervals, during the 10 min preceding ingestion (pre-ingestion period); (b) during the meal consumption (ingestion period), following each meal serving; and (c) after the meal (postprandial period), initially at 5 min intervals for the first 20 min, and subsequently at 10 min intervals up to 60 min following the end of ingestion (as illustrated in Figure 1). These scales have proven to detect differences induced by various conditioning mechanisms with a high degree of consistency and replicability; additionally, it has been shown that perception scores correlate with levels of circulating metabolites and with indicators of brain function [1].

### 2.6. Statistical Analysis

On each study day, we evaluated sensation scores by examining the AUC, which was adjusted for baseline values with the exception of the wanting and liking measures, which were not adjusted. The calculation procedure involved the following steps: for each observation period, the area was computed by multiplying the duration (measured in minutes) of that period by the adjusted score (the absolute score minus the mean score before the meal). To derive the overall AUC (expressed as a product of score and minutes), the sum of the areas from all observation periods was determined.

In each participant, the impact of the high-fat meal was calculated as the difference in the AUC on the second day (high-fat meal) minus the first day (regular meal); the effect of aversive conditioning was quantified as the difference between the third day (regular meal after previous conditioning) and the first day (regular meal preconditioning; day 3 minus day 1), and the effect of the cognitive intervention was quantified as the difference between the fourth day (postintervention) and the third day (aversive conditioning; day 4 minus day 3). For graphic representation of the data, average values and standard errors for the recorded variables within each group were calculated and are presented in the figures below.

We conducted statistical analyses both within each group and between the groups. The normality of data distribution was determined using the Shapiro–Wilk test, which determined the normal distribution of data. For normally distributed data, we employed Student’s *t*-test (either paired or unpaired). In cases where the data distribution was not normal, paired data were analysed using the Wilcoxon signed-rank test and unpaired data using the Mann–Whitney U test.

A sensitivity analysis to control for individual variations and confounders whilst obviating the need for artificial divisions between normally and nonnormally distributed data was included. The data were analysed for each day of the experiment, treatment groups were compared on the sensation score outcomes using generalized linear mixed modelling (GLMM), with time (in minutes), intervention group and time—intervention group interaction terms included. To take into account the correlation between each individual patient’s responses over time, we included the individual as a random effect. Visual inspections of residual plots were used to determine deviations from normality. In the absence of normality, robust variance estimators were calculated.

The SPSS Statistics package for Windows (Version 28.0, IBM Corp., Armonk, NY, USA) was used for all calculations (significance level of 5%, two-tailed), except the generalized lineal mixed models, which were calculated using R studio version 4.1.2.

All co-authors reviewed the data and provided approval for the final version of the manuscript.

## 3. Results

### 3.1. Demographics and Study Flow

The average age of participants was 29.9 ± 2.7 years of age (range 19–44 years). Their body mass index averaged at 21.3 ± 0.5 kg/m^2^ (range 19.8–23.9). On the physical anhedonia scale, their average score was 15.3 ± 1.7 in the (range 6–24) all were non-smokers. Baseline characteristics were not different between groups. All participants adhered to the instructions, completed the study protocol, and were analysed.

### 3.2. Original Responses to the Comfort Meal (Study Day 1)

**Figure 2:**
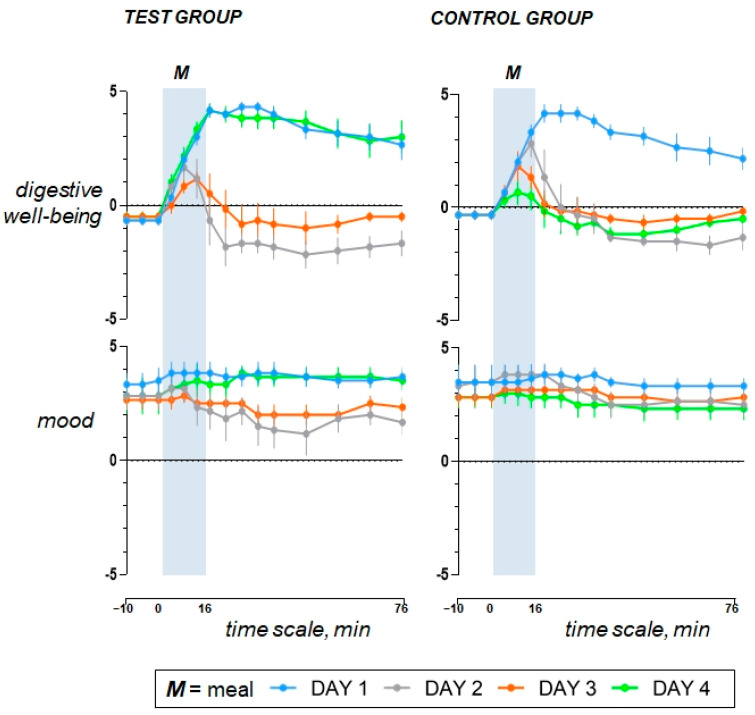
Hedonic sensations. The comfort meal on day 1 was associated with a gratifying postprandial experience, which was significantly reduced after aversive conditioning on day 3 mimicking the response to the offending meal on day 2; this effect reverted by the cognitive intervention on day 4, thereby restoring the hedonic value of the comfort meal. Aversive conditioning (i.e., the difference in the AUC on day 3 minus day 1) was equivalent between groups. The effect of the cognitive intervention (i.e., the difference in the AUC on day 4 minus day 3) was different than that of the sham intervention in the control group. Values represent mean ± SE.

**Figure 3:**
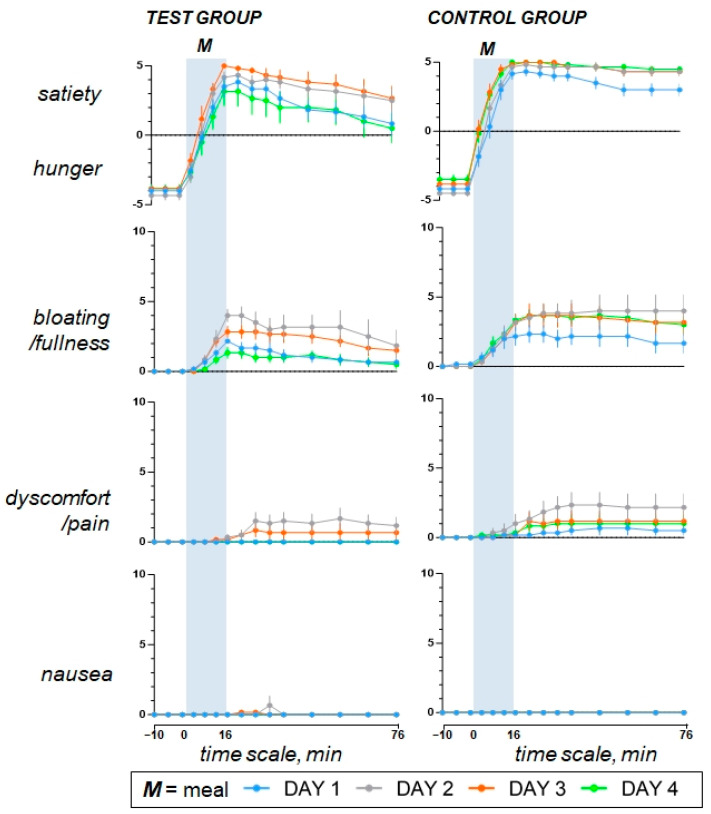
Homeostatic sensations. Aversive conditioning was associated with an increase in satiety, fullness/bloating, and mild abdominal discomfort. No differences between groups were detected for the effects of aversive conditioning (i.e., the difference in the AUC on day 3 minus day 1) or the cognitive intervention (i.e., the difference in the AUC on Day 4 minus Day 3) for satiety, bloating, discomfort, or nausea. Values represent mean ± SE.

**Figure 4:**
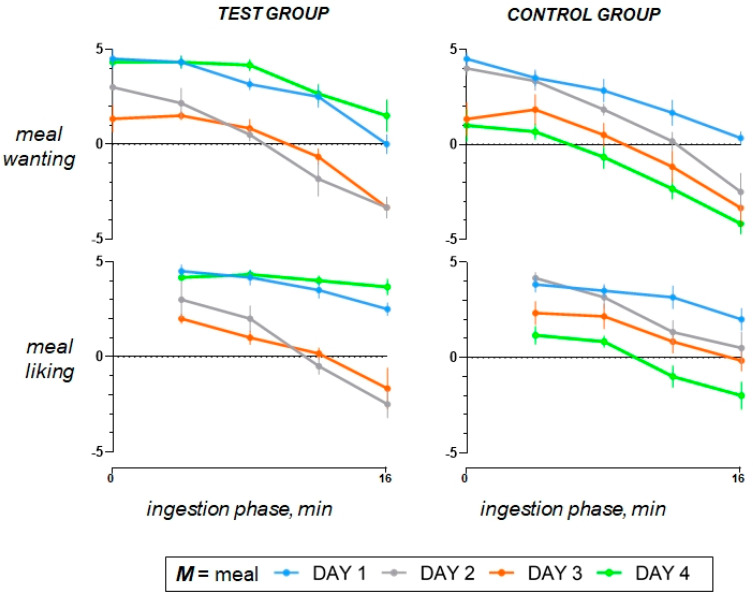
Meal valence during the ingestion phase. The administration of the comfort meal was carried out in a stepwise manner, divided into four equal servings; meal wanting was measured before each serving and at the end of ingestion; meal liking was measured after each serving. On day 1, participants enjoyed the comfort meal, but meal wanting and liking were significantly reduced after aversive conditioning on day 3; this effect reverted by the cognitive intervention on day 4, thereby restoring the reward value of the comfort meal. The effect of aversive conditioning (i.e., the difference in the AUC on day 3 minus day 1) was equivalent between groups. The effect of the cognitive intervention (i.e., the difference in the AUC curve on day 4 minus day 3) was significantly different from that of the sham intervention in the control group. Values represent mean ± SE.

During the pre-ingestion period, participants scored in the questionnaires a sensation of hunger with positive mood, neutral digestive wellbeing, and no sensation of abdominal fullness/bloating, nausea, or discomfort/pain (Figure 2 and Figure 3). Participants liked the comfort meal (regular-fat non-coloured hummus) and rated meal wanting and meal liking with positive scores (Figure 4). As the meal was consumed, satiety gradually increased and was accompanied by a mild sense of fullness, positive mood, and a marked increase in digestive wellbeing, without symptoms such as nausea or abdominal discomfort (Figure 2 and Figure 3). During the postprandial period, the intensity of these sensations gradually decreased (Figure 2 and Figure 3). The responses to the comfort meal were not different between groups.

### 3.3. Offending Meal (Study Day 2 versus Day 1)

The fat overload did not affect palatability of the offending meal (high-fat coloured hummus), and at initial taste, meal liking was rated similar as the comfort meal (*p* = 0.491); however, with further ingestion, meal liking and wanting decayed (*p* < 0.001 for both; Figure 4). As compared to the comfort meal, completion of the high-fat meal resulted in more satiety, more fullness/bloating, and mild discomfort/pain, but no nausea, with markedly impaired digestive wellbeing and mood during the postprandial period (Figure 2 and Figure 3).

### 3.4. Aversive Conditioning (Study Day 3 versus Day 1)

Following the consumption of the high-fat coloured meal (offending meal) the day prior, both the meal wanting and meal liking of the regular-fat meal, labelled with the same colour, noticeably declined in comparison to the preconditioning exposure to the same meal on the initial study day (*p* < 0.001 for both; Figure 4). After conditioning, ratings of the regular-fat meal mimicked those of the offending high-fat meal, and compared to the responses to the original comfort meal, aversive conditioning significantly impaired postprandial wellbeing (*p* < 0.001). It had lesser impact on mood (*p* = 0.386), and resulted in increased homeostatic sensation, particularly satiety (*p* = 0.008) and fullness/bloating (*p* = 0.010) accompanied by mild abdominal discomfort (0.003) and without nausea (Figure 2 and Figure 3). Aversive conditioning (quantified as the change in the AUC curve on day 3 minus day 1) had a similar effect in both groups: digestive wellbeing (change by −260 ± 53 vs. −211 ± 28 score × min in controls; *p* = 0.435), mood (change by −42 ± 36 vs. 4 ± 18 score × min in controls; *p* = 0.248), satiety (change by 92 ± 41 vs. 48 ± 25 score × min in controls; *p* = 0.589), bloating/fullness (change by 72 ± 34 vs. 92 ± 31 score × min in controls; *p* = 0.678), abdominal discomfort (change by 40 ± 35 vs. 39 ± 29 score × min in controls; *p* = 1.000), nausea (change by 2 ± 2 vs. 0 ± 0 score × min in controls; *p* = 0.699).

### 3.5. Effect of the Cognitive Intervention (Day 4 versus Day 3)

The cognitive intervention reverted the effects of aversive conditioning and restored the original responses to the comfort meal observed at initial exposure on study day 1 (Figure 2 and Figure 3). Indeed, as compared to study day 3, the cognitive intervention increased meal wanting and liking, improved digestive wellbeing and mood, tended to reduce postprandial satiety, bloating/fullness, and abolished discomfort/pain in response to the original comfort meal (regular-fat non-coloured hummus) consumed on study day 4 (Figure 2, Figure 3 and Figure 4). By contrast, sham intervention in the control group had no effects, and on repeat exposure to the regular-fat meal, without colour labelling on the fourth study day, the postprandial sensations remained like the responses to the offending meal (Figure 2 and Figure 3), except for meal wanting and meal liking, which declined further (*p* = 0.013 and 0.024, respectively) (Figure 4). The effect of the cognitive intervention (i.e., the change in the AUC on day 4 minus day 3) was significantly different from that of the sham intervention in the control group for meal wanting (change by 60 ± 9 vs. −18 ± 5 score × min in controls; *p* < 0.001), meal liking (change by 59 ± 8 vs. −25 ± 6 score × min in controls; *p* < 0.001), digestive wellbeing (change by 249 ± 52 vs. −25 ± 17 score × min in controls; *p* < 0.001), and mood (change by 75 ± 31 vs. −27 ± 21 score × min in controls; *p* = 0.023), and the differences were not statistically significant for satiety (change by −120 ± 44 vs. −14 ± 7 score × min in controls; *p* = 0.132), fullness/bloating (−64 ± 31 vs. 1 ± 34 score × min in controls; *p* = 0.192), abdominal discomfort (change by −40 ± 35 vs. −11 ± 12 score × min in controls; *p* = 0.485), or nausea (change by −2 ± 2 vs. 0 ± 0 score × min in controls; *p* = 0.699).

### 3.6. Results of the Sensitivity Analysis Using Generalized Linear Mixed Modelling (GLMM)

Overall, the results of the sensitivity analysis confirmed the effect of the intervention in the same direction for all the outcomes evaluated. Significant differences in the responses between groups were detected only during the fourth day of the experiment. The full analysis is presented in Table 1 for hedonic sensation, in Table 2 for homeostatic sensations, and in Table 3 for meal valence. No significant nausea was scored during the experiments; thus, no sensitivity analysis was performed for this outcome.

## 4. Discussion

Our proof-of-concept study demonstrates that a cognitive intervention, under certain circumstances, may revert conditioned food aversion, and restore the reward value of an originally pleasant meal in healthy women.

The probe meal in the present study, a tasty hummus, induced the pleasant response characteristic of a comfort meal. Aversive conditioning was achieved by administration of the same hummus containing a hidden fat overload that did not affect its organoleptic characteristics, specifically the palatability, but induced an aversive postprandial response. After this experience, subjects became conditioned and reacted to the original comfort meal mimicking their response to the offending meal. In the test group, the cognitive intervention reverted conditioning through an unlearning process and restored the original response to the comfort meal. By contrast, in the control group, the response to the originally comfort meal, particularly meal wanting and liking, further deteriorated through re-exposure, suggesting that some degree of aversive reinforcement took place.

Taste aversion and aversive conditioning of the postprandial experience, both in nonhuman animals and in humans, as in the present study, can be acquired after a single conditioning trial [3,14] in contrast to other forms of Pavlovian conditioning, which require repeat pairing experiences [5]. In nonhuman animals, this hedonic shift endures and continues to remain until the food is experienced repeatedly without ensuing illness [2]. However, our study suggests that the situation may be different in humans, because a single cognitive intervention reverted conditioning.

Disclosing the prior conditioning paradigm, and realization of the true situation, resulted in unlearning and reversion of conditioning. We have previously shown that, using a cognitive–sensory intervention, healthy subjects learn to heighten their hedonic response to a comfort meal [15]. Interestingly, learning not only stimulated the gustative experience, but more so the postprandial response. Education has also proven effective in overcoming neophobia in children, i.e., the natural tendency to reject new or unknown foods [16,17].

Based on previous experience [18], the probe meal involved a tasty hummus, which allows for relevant changes in composition without impact on the organoleptic characteristics. Indeed, the fat overload of the offending meal did not affect its palatability, measured using the initial liking score, but induced a metered aversive postprandial experience. As a result, conditioning by exposure to this meal blunted the postprandial hedonic response to the comfort meal, but it did not affect homeostatic sensations, and did not induce nausea or abdominal pain; furthermore, conditioning reduced meal liking but did not induce real taste aversion. It remains uncertain whether cognitive intervention may revert stronger food aversions with homeostatic or emotive components, which are more resistant to extinction [19,20].

Our conditioning paradigm involved a colour clue (non-coloured meals before conditioning on day 1 and after the intervention on day 4, in contrast to colour-labelling of the offending meal on day 2 and postconditioning meal on day 3), but we cannot ascertain whether the colour clue influenced the effect of the intervention. However, in the control group, the response to the regular-fat meal on the fourth day induced an aversive response even without the colour label, indicating that conditioning was not colour-specific. It has been previously shown that other forms of conditioning affect related stimuli by a phenomenon of generalization [19,21].

An important contribution of our study is the focus on the postprandial experience over taste. Taste aversion in nonhuman animals function as a protective mechanism to prevent ingestion of potentially noxious foods [2,3,14]; however, in humans, the postprandial impact seems more relevant, as indicated by the common experience of tasty foods that feel bad, and despite being aware of it, palatability is not impaired.

## 5. Limitations

Due to the exploratory nature and complexity of the study, the sample size was small, and in line with previous study, only women were included, as they are more susceptible to conditioning. Additionally, the cognitive intervention was tested on a relatively mild aversive conditioning model and was applied shortly after the acquisition of aversion. Therefore, as a proof-of-concept study, these results need to be interpreted with caution. Our findings cannot be generalized to demonstrate the effectiveness of the cognitive intervention on a larger population, including men, and on stronger and longer-lasting food aversions remains to be established.

## 6. Conclusions and Inferences

The relevance of our study lies in the potential application of unlearning aversive conditioning in clinical practice. Symptoms in patients with functional digestive disorders may originate from aversive conditioning, leading to hypersensitivity and hypervigilance [5,7,8,9], and unlearning could provide a mechanistic treatment strategy. The intervention to prevent postprandial symptoms may require, as in nonhumans, more elaborate paradigms, including a sensory component and repeat exposures pairing meals with non-noxious or pleasant sensations [21].

## Figures and Tables

**Table 1:** Generalised linear mixed model for hedonic sensations.

**Dependent Variable:**	**Digestive Well-Being**			
	**Day 1**	**Day 2**	**Day 3**	**Day 4**
Group (intervention)	−0.169 ± 0.226	−0.832 * ± 0.435	−0.311 ± 0.317	1.949 *** ± 0.394
Time	0.031 *** ± 0.01	−0.033 *** ± 0.009	−0.009 *** ± 0.003	−0.011 * ± 0.006
Group×time	0.015 ± 0.014	0.005 ± 0.014	0.001 ± 0.006	0.056 *** ± 0.015
Constant	1.813 *** ± 0.174	0.411 ± 0.34	0.156 ± 0.244	−0.222 ± 0.337
Observations	180	180	180	180
Log likelihood	−367.151	−356.12	−294.939	−325.659
Akaike inf. crit.	746.302	724.24	601.878	663.318
Bayesian inf. crit.	765.46	743.398	621.036	682.476
**Dependent Variable:**	**Mood**			
	**Day 1**	**Day 2**	**Day 3**	**Day 4**
Group (intervention)	0.017 ± 0.689	−1.092 ± 0.859	−0.496 ± 0.341	0.373 ± 0.755
Time	−0.005 ± 0.007	−0.019 * ± 0.011	−0.005 ± 0.004	−0.010 *** ± 0.003
Group×time	0.006 ± 0.007	0.011 ± 0.013	−0.003 ± 0.007	0.021 ** ± 0.009
Constant	3.622 *** ± 0.536	3.506 *** ± 0.513	3.044 *** ± 0.205	2.830 *** ± 0.441
Observations	180	180	180	180
Log likelihood	−163.442	−254.835	−164.307	−184.011
Akaike inf. crit.	338.884	521.67	340.614	380.023
Bayesian inf. crit.	358.042	540.827	359.772	399.18

* *p* < 0.1; ** *p* < 0.05; *** *p* < 0.01.

**Table 2:** Generalised linear mixed model for homeostatic sensations.

**Dependent Variable:**	**Satiety**			
	**Day 1**	**Day 2**	**Day 3**	**Day 4**
Group (intervention)	−0.278 ± 0.478	−0.348 ± 0.301	−0.452 * ± 0.261	−1.477 ** ± 0.641
Time	0.096 *** ± 0.007	0.122 *** ± 0.007	0.106 *** ± 0.005	0.107 *** ± 0.007
Group×time	−0.033 * ± 0.019	−0.025 * ± 0.015	−0.015 ± 0.012	−0.045 *** ± 0.016
Constant	−0.162 ± 0.362	−0.026 ± 0.122	0.740 *** ± 0.149	0.787 *** ± 0.185
Observations	180	180	180	180
Log likelihood	−450.093	−457.832	−455.353	−444.794
Akaike inf. crit.	912.186	927.664	922.707	901.589
Bayesian inf. crit.	931.344	946.822	941.865	920.746
**Dependent Variable:**	**Bloating/Fullness**			
	**Day 1**	**Day 2**	**Day 3**	**Day 4**
Group (intervention)	−0.349 ± 0.298	0.083 ± 0.314	−0.234 ± 0.329	−1.153 *** ± 0.245
Time	0.022 ** ± 0.01	0.063 *** ± 0.019	0.048 *** ± 0.014	0.046 *** ± 0.011
Group×time	−0.016 ± 0.01	−0.03 ± 0.027	−0.025 ± 0.016	−0.022 * ± 0.012
Constant	1.144 *** ± 0.272	1.480 *** ± 0.232	1.487 *** ± 0.264	1.595 *** ± 0.163
Observations	180	180	180	180
Log likelihood	−275.225	−374.271	−337.335	−300.091
Akaike inf. crit.	562.45	760.542	686.669	612.183
Bayesian inf. crit.	581.608	779.7	705.827	631.34
**Dependent Variable:**	**Discomfort/Pain**			
	**Day 1**	**Day 2**	**Day 3**	**Day 4**
Group (intervention)	−0.093 ± 0.061	0.896 ** ± 0.395	−0.112 ± 0.192	−0.287 ± 0.181
Time	0.01 ± 0.007	0.048 *** ± 0.015	0.020 * ± 0.012	0.016 * ± 0.01
Group×time	−0.01 ± 0.007	−0.016 ± 0.024	−0.008 ± 0.016	−0.016 * ± 0.01
Constant	0.093 ± 0.061	0.668 ** ± 0.334	0.301 * ± 0.163	0.287 ± 0.181
Observations	180	180	180	180
Log likelihood	−85.033	−357.008	−247.848	−162.538
Akaike inf. crit.	182.066	726.016	507.697	337.076
Bayesian inf. crit.	201.224	745.174	526.855	356.233

* *p* < 0.1; ** *p*< 0.05; *** *p* < 0.01.

**Table 3:** Generalised linear mixed model for meal valence.

**Dependent Variable:**	**Wanting**			
	**Day 1**	**Day 2**	**Day 3**	**Day 4**
Group (intervention)	0.467 ± 0.482	−1.167 ± 0.911	−0.067 ± 0.962	3.300 *** ± 0.394
Time	−0.254 *** ± 0.031	−0.404 *** ± 0.053	−0.308 *** ± 0.083	−0.333 *** ± 0.006
Group×time	−0.017 ± 0.037	−0.013 ± 0.101	0.021 ± 0.099	0.150 *** ± 0.015
Constant	4.600 *** ± 0.38	4.600 *** ± 0.302	2.300 *** ± 0.871	1.567 *** ± 0.337
Observations	60	60	60	60
Log likelihood	−90.783	−115.857	−119.504	−104.623
Akaike inf. crit.	193.566	243.714	251.009	221.247
Bayesian inf. crit.	206.132	256.28	263.575	233.813
**Dependent Variable:**	**Wanting**			
	**Day 1**	**Day 2**	**Day 3**	**Day 4**
Group (intervention)	0.75 ± 0.688	−0.25 ± 1.24	−0.167 ± 0.991	1.917 *** ± 0.628
Time	−0.146 *** ± 0.054	−0.321 *** ± 0.061	−0.221 *** ± 0.048	−0.283 *** ± 0.063
Group×time	−0.021 ± 0.059	−0.154 ± 0.104	−0.075 ± 0.095	0.237 *** ± 0.069
Constant	4.583 *** ± 0.533	5.500 *** ± 0.514	3.500 *** ± 0.782	2.583 *** ± 0.582
Observations	48	48	48	48
Log likelihood	−70.431	−87.037	−82.738	−68.674
Akaike inf. crit.	152.863	186.074	177.475	149.347
Bayesian inf. crit.	164.09	197.302	188.703	160.575

*** *p* < 0.01.

## Data Availability

The data presented in this study will be shared upon reasonable request from the corresponding author.
